# Maize Genotypes With Different Zinc Efficiency in Response to Low Zinc Stress and Heterogeneous Zinc Supply

**DOI:** 10.3389/fpls.2021.736658

**Published:** 2021-10-08

**Authors:** Jianqin Xu, Xuejie Wang, Huaqing Zhu, Futong Yu

**Affiliations:** Key Laboratory of Plant-Soil Interaction (MOE), Centre for Resources, Environment and Food Security, College of Resources and Environmental Sciences, China Agricultural University, Beijing, China

**Keywords:** maize, Zn deficiency, heterogeneous Zn supply, root plasticity, *ZIP* genes

## Abstract

All over the world, a common problem in the soil is the low content of available zinc (Zn), which is unevenly distributed and difficult to move. However, information on the foraging strategies of roots in response to heterogeneous Zn supply is still very limited. Few studies have analyzed the adaptability of maize inbred lines with different Zn efficiencies to different low Zn stress time lengths in maize. This study analyzed the effects of different time lengths of low Zn stress on various related traits in different inbred lines. In addition, morphological plasticity of roots and the response of Zn-related important gene iron-regulated transporter-like proteins (ZIPs) were studied via simulating the heterogeneity of Zn nutrition in the soil. In this report, when Zn deficiency stress duration was extended (from 14 to 21 days), under Zn-deficient supply (0.5 μM), Zn efficiency (ZE) based on shoot dry weight of Wu312 displayed no significant difference, and ZE for Ye478 was increased by 92.9%. Under longer-term Zn deficiency, shoot, and root dry weights of Ye478 were 6.5 and 2.1-fold higher than those of Wu312, respectively. Uneven Zn supply strongly inhibited the development of some root traits in the -Zn region. Difference in shoot dry weights between Wu312 and Ye478 was larger in T1 (1.97 times) than in T2 (1.53 times). Under heterogeneous condition of Zn supply, both the –Zn region and the +Zn region upregulated the expressions of *ZmZIP3, ZmZIP4, ZmZIP5, ZmZIP7*, and *ZmZIP8* in the roots of two inbred lines. These results indicate that extended time length of low-Zn stress will enlarge the difference of multiple physiological traits, especially biomass, between Zn-sensitive and Zn-tolerant inbred lines. There were significant genotypic differences of root morphology in response to heterogeneous Zn supply. Compared with split-supply with +Zn/+Zn, the difference of above-ground biomass between Zn-sensitive and Zn-tolerant inbred lines under split-supply with –Zn/+Zn was higher. Under the condition of heterogeneous Zn supply, several *ZmZIP* genes may play important roles in tolerance to low Zn stress, which can provide a basis for further functional characterization.

## Introduction

Zinc (Zn) is an essential micronutrient in plant growth and development. It plays an important role in various enzymatic reactions, metabolic processes, redox reactions, plant hormone metabolism, promoting the development of plant reproductive organs, resistance to infection by certain pathogens, and improving plant resistance to stress (Shemi et al., 2021; Suganya et al., 2021). But because of the adsorption and fixation of calcium carbonate, organic matter, phosphate, and clay in the soil, the effectiveness of Zn in the soil is low (Cakmak, 2008). Zn deficiency is probably the most prevalent micronutrient deficiency in soils (Rehman et al., 2021). The lack of Zn in the soil leads to lower yields (Aziz et al., 2017), and affects nutritional quality of crop plants (Cakmak and Kutman, 2018). Zn deficiency in plants causes damage to plant cells mainly at the cell membrane level (Candan et al., 2018) and can also alter mitochondrial ultrastructure (Chen et al., 2009).

Approximately 50% of the arable land for food production is in Zn deficiency (Yu et al., 2020), which contributes to plant and human Zn malnutrition (Shahzad et al., 2014). According to a report from the WHO (2016), around 30% of the world's population is Zn-deficient. Particularly, it is prevalent in developing countries (Guo et al., 2020). The insufficient absorption of such micronutrient leads to many serious health problems (Montoya et al., 2021) such as depression, psychosis, pneumonia, diarrhea, impaired physical and/or neural development, decreased immune-competence, and increased rates of infectious diseases (Manwaring et al., 2016). Deficiency of Zn is also prominent in pregnant women and therefore, causes infant mortality (Ganie et al., 2020). In addition, deaths of children under the age of five caused by Zn deficiency can reach 116,000 a year (Rehman et al., 2021). Therefore, the improvement of the trace element Zn deficiency is very important for animals, plants, and humans (Zhou et al., 2020).

Maize has a great significance as a source of food, animal feed, and raw material for various industrial products. China is the second largest corn producer in the world and is responsible for 22% of global maize output from 2012 to 2014 (FAOSTAT, 2017). The cultivated area for maize in China is estimated to be 42.42 million ha with yield of about 259.23 million tons year^−1^ (Shemi et al., 2021). Compared with other crops, maize has a high requirement for Zn and, thus, is known as an indicator plant for the evaluation of Zn deficiency of soil in an area (Zhang et al., 2021).

There are significant differences among plant species in their response to foliar Zn sprays. In increasing grain Zn concentration, wheat was the most responsive crop to leaf Zn spray (up to 83%), followed by rice (up to 27%) and maize (9%) (Cakmak and Kutman, 2018). Zn deficiency symptoms in maize at the seedling stage can be used to identify efficient genotypes and in routine screening for Zn efficiency (Genc et al., 2002).

Many studies have proposed that the most sensitive and effective evaluation parameters for the difference in Zn efficiency between different genotypes are as follows: dry matter production, visual symptoms of the severity of Zn deficiency, R/S ratio, Zn content, and Zn uptake efficiency. On the other hand, parameters that have poor correlation with Zn efficiency includes Zn concentration and differences of subcellular Zn compartmentation (Rengel and Graham, 1996; Cakmak et al., 1997, 2001; Grewal et al., 1997; Khan et al., 1998; Erenoglu et al., 1999; Grewal and Williams, 1999; Hacisalihoglu and Kochian, 2003; Hacisalihoglu et al., 2004; Genc et al., 2006; Sadeghzadeh et al., 2009; Impa et al., 2013a).

Combining agronomic, ecological and economic factors, and the development and use of high-efficiency Zn varieties is an effective and sustainable method to solve the problem of Zn deficiency in maize.

The Zn-regulated transporters and the iron-regulated transporter-like proteins are considered to be the primary group of transporters controlling plant Zn influx (Eide, 2006). They are suggested to play critical roles in balancing Zn homeostasis. Most ZIPs are predicted to have similar membrane topologies and eight transmembrane domains with their C and N-termini located on the outside surface of plasma membrane (Durmaz et al., 2011). ZmZIP proteins share a conserved transmembrane domain and a variable region between TM-3 and TM-4 (Li et al., 2013). The response of *ZIP* genes to different Zn concentrations differs between members, but most *ZIP* genes reported are upregulated by Zn deficiency (Yang et al., 2009). Moreover, some of the *ZIP* family members are constitutively expressed. The expression sites of *ZIP*s in plants and their affinity for Zn ions may be different, and their expression levels are also affected by the concentration of metal ions in the growth medium (Assunção et al., 2010).

In *Arabidopsis*, there are 16 *ZIP* genes, and approximately half of the *ZIP* genes are induced in response to Zn deficiency (Lin et al., 2009). ZIP1- ZIP4 proteins functionally complement a yeast strain defective in Zn uptake (Grotz and Guerinot, 2006). Transcript levels of several *ZIP*s, such as *AtZIP1* to *AtZIP5*, and *AtZIP9* to *AtZIP12* are increased under Zn-limiting conditions in roots and/or shoots of *Arabidopsis* (Lee et al., 2010). The *Arabidopsis thaliana* basic-region leucine-zipper (bZIP) transcription factor gene family, bZIP19 and bZIP23, contributes to the upregulation of *ZIP*s and improves the adaptation to low Zn supply (Assunção et al., 2010). HvbZIP56 and HvbZIP62 partially rescue the Zn-dependent growth phenotype and ZIP-transporter gene regulation of an *Arabidopsis bzip19-4 bzip23-2* mutant. MtZIP1, MtZIP5, and MtZIP6 proteins restore yeast growth on Zn-limited media in the model legume *Medicago truncatula* (Lopéz-Millán et al., 2004). Six *HvZIP* genes (*HvZIP3*, -*5*, -*7*, -*8*, -*10*, -*13*) are highly induced in roots of Zn-deficient plants. Tissue-specific expression in roots supports the roles of these genes in uptake and root-to-shoot translocation of Zn under Zn starvation conditions (Tiong et al., 2015). Some of the rice *ZIP*s are also induced by Zn deficiency, including *OsZIP1, OsZIP3, OsZIP4, OsZIP5*, and *OsZIP8* (Ramesh et al., 2003; Ishimaru et al., 2005; Lee et al., 2010). However, so far, there is still a lack of detailed analysis of the response of *ZIP*s to low Zn stress in different lines as the results of some studies are inconsistent. For example, Ramesh et al. (2003) considered that OsZIP1, OsZIP3 were rice Zn transporters induced by Zn deficiency, and expressed in the vascular bundles. However, Ishimaru et al. (2005) suggested that expression of these two genes was induced by Cu stress, rather than Zn deficiency.

In the process of crop production, excessive input of fertilizer will lead to waste of resources. Local application of fertilizers can promote the absorption and utilization of nutrient elements by field crops. However, under the condition of local nutrient supply, it is difficult to study the response of crops to this nutrient due to the complexity of soil influencing factors. In addition, the spatial and temporal distribution of resources in the soil is cohesive and heterogeneous (Hodge, 2006). To compete for the resources of nutrient-enriched patches, plant roots show morphological and physiological plasticity to meet their own nutrient requirements (Hodge, 2004). Different plants have selective carbon allocation strategies for heterogeneous nutrient distribution including changes in total root length, root biomass, lateral root density, specific root length, and unit lateral root length (Guo et al., 2005). In the soil solution, Zn is affected by soil pH, organic matter, and other elements which makes the distribution and availability of Zn heterogeneous. Due to poor mobility of Zn, the morphological plasticity of root system may be more important for heterogeneous Zn nutrition than the physiological response. To our limited knowledge, under the condition of heterogeneous Zn supply, the research of root system related genes and morphological response has not yet been reported. Related studies have analyzed the changes in plant physiological traits and root morphology under heterogeneous phosphorus and nitrogen conditions (Yu et al., 2015, 2016; Liu et al., 2020). But when dealing with the heterogeneous supply of Zn, what changes will the plant physiological characteristics, and related genes show? Does it respond to Zn-rich and Zn-deficient patches through adaptive morphological changes and show carbon-saving strategies?

Therefore, we set up different Zn supply levels to analyze the reasons for the differences in Zn efficiency of different genotypes. Under the condition of heterogeneous Zn nutrient supply, the internal control mechanism of maize tolerance to low-Zn stress was analyzed by studying the response of maize roots to Zn deficiency and the expression of *ZIP* family genes induced by Zn deficiency.

## Materials and Methods

### Seed Germination and Hydroponic Culture

Maize seeds of inbred lines Wu312 and Ye478 were sterilized for 30 min in a 10% solution of H_2_O_2_, washed with distilled water and soaked in saturated CaSO_4_ for 10 h, and then germinated on moist filter paper in the dark at room temperature. Two days later, the germinated seeds were wrapped in moist filter paper roll and grown. At the stage of two visible leaves, the seedlings were transferred into a full-strength nutrient solution with the following composition (mM): 0.5 NH_4_NO_3_, 0.5 CaCl_2_, 1.5 Ca(NO_3_)_2_, 0.75 K_2_SO_4_, 0.65 MgSO_4_, 0.1 KCl, 0.25 KH_2_PO_4_, 1.0 × 10^−3^ H_3_BO_3_, 0.35 Fe(II)-EDTA, 8.0 × 10^−3^ CuSO_4_, 1.2 × 10^−2^ MnSO_4_, 4.0 × 10^−5^ (NH_4_)Mo_7_O_24_, and 4.0 × 10^−3^ NiCl. Growth chamber condition was set as a 14-h light period from 8:00 to 22:00 with 28°C and a 10 h dark period with 22°C. The average light intensity measured at canopy was 350 μmol m^−2^ s^−1^. The pH of solution was adjusted to 5.5–6.0.

### Experiment Design

Zn deficiency in crops is usually corrected through the application of ZnSO_4_·7H_2_O. However, a study showed that compared with ZnSO_4_, ethylene diamine tetra acetic acid-Zn (EDTA-Zn) was found to be better for growth and yield of rice (Naik and Das, 2007). In addition, many studies have found that EDTA-Zn contributes to the increase in Zn-use efficiency more than ZnSO_4_·7H_2_O. In addition, there is approximately a 3:1–5:1 advantage for EDTA-Zn in comparison with ZnSO_4_ (Hegert et al., 1984; Modaihsh, 1990; Alvarez et al., 1996; Martín-Ortiz et al., 2009; Ghasal et al., 2018). Therefore, we chose to use EDTA-Zn in this research.

**Figure 1:**
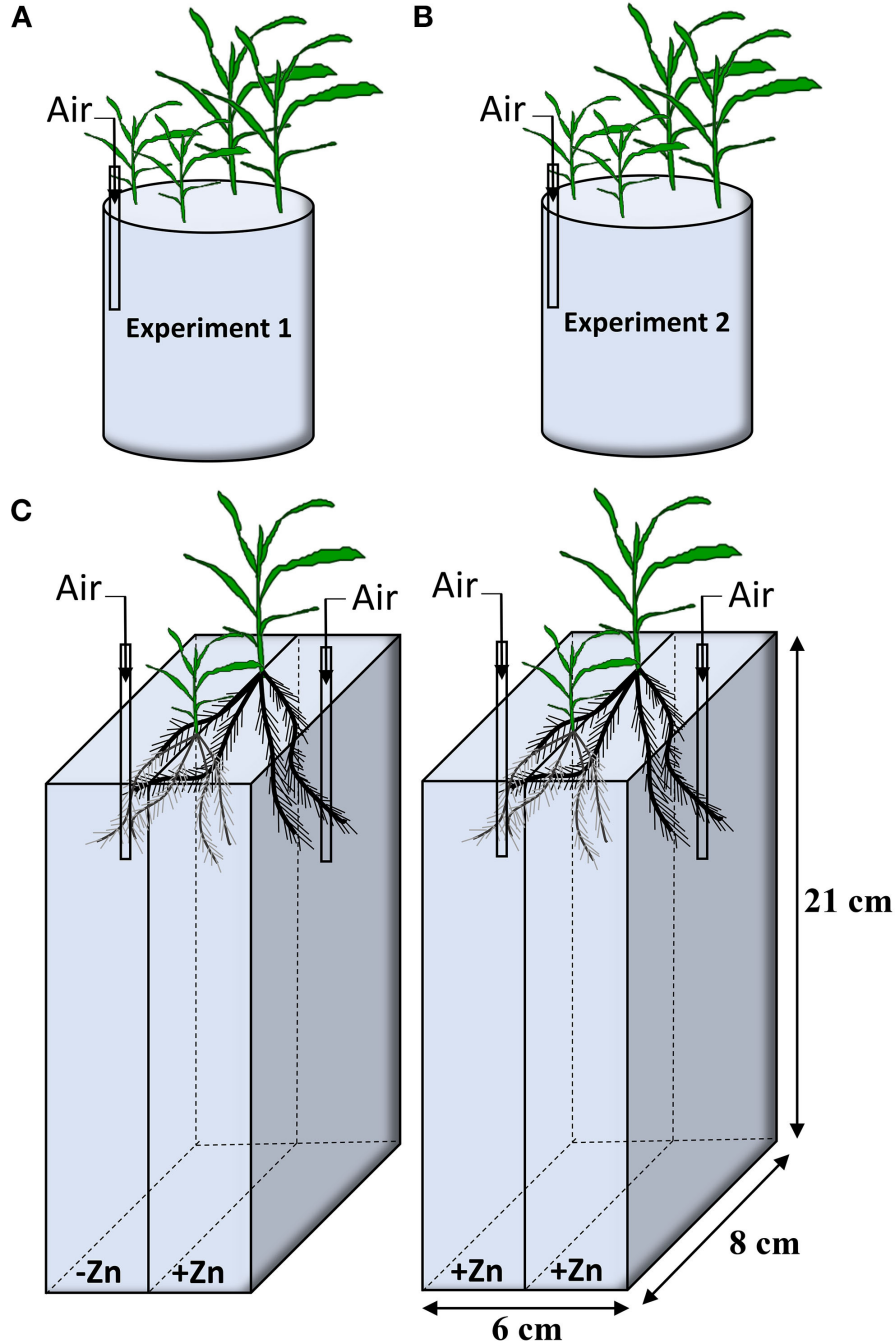
Experiment design in this study. **(A)** In Experiment 1, two plants of inbred lines Wu312 and Ye478 were mix-cropped in a 3.3-liter container. Seven different EDTA-Zn levels (0, 0.05, 0.1, 0.2, 0.4, 0.5, 8 μM) were set up. **(B)** In Experiment 2, two seedlings of each inbred line were grown at eight different Zn nutritional status (0, 0.5, 1, 2, 4, 8, 16, 32 μM EDTA-Zn). **(C)** Two-compartment container was used in Experiment 3. Each seedling of Wu312 and Ye478 was grown hydroponically in mix-cropping under split Zn supply [0.5 μM for Zn-deficient condition (–Zn) and 8 μM for Zn-sufficient condition (+Zn)]. There were three replicates in Experiment 1 and 2, and four replicates in Experiment 3. Each experiment was continuously aerated.

In Experiment 1, two seedlings for Wu312 and Ye478 were mix-cropped in a 3.3 L container at eight Zn nutritional status in the growth chamber. Seven Zn levels were set up, including 0, 0.05, 0.1, 0.2, 0.4, 0.5, 8 μM EDTA-Zn (Figure 1A). In Experiment 2, two seedlings for each inbred line were hydroponically grown at different Zn nutritional status. Eight treatments supplied with EDTA-Zn were design, containing 0, 0.5, 1, 2, 4, 8, 16, 32 μM (Figure 1B). The nutrient solution was continuously aerated and renewed every 3 days. Each treatment contained three replicates in Experiment 1 and 2. Plants of Experiment 1 and 2 were harvested 14 and 21 days after transfer, respectively. Shoot and root dry weight, R/S ratio, Zn concentration, and content in shoot and root of each sample were measured, and Zn uptake, transport and use efficiency of each plant were estimated.

Experiment 3 was designed as a 21-day split-root experiment which included two treatments. Plants of Wu312 and Ye478 were grown under split-supply with -Zn (0.5 μM EDTA-Zn) and +Zn (8 μM EDTA-Zn) in Treatment 1 (T1), and with +Zn (8 μM EDTA-Zn) and +Zn (8 μM EDTA-Zn) in Treatment 2 (T2), respectively (Figure 1C). After 4 days of hydroponic culture using full-strength solution, seminal roots for each seedling was cut out, and the remaining roots were evenly divided into two parts and transferred into a black two-compartment container (21-cm long, 6-cm wide, 8-cm high). There were two slots evenly distributed along the wall in the middle of the compartments and one seedling of each inbred line was separately fixed with sponge strips in one slot within the same container. Each compartment contained 1.5 L of nutrient solution. The nutrient solution was continuously aerated and renewed every 3 days. Each treatment included four replicates in Experiment 3. Plants in Experiment 3 were harvested at 21 days after transfer. Six root traits of each sample were determined, including total root length, root surface, tap root length, specific root length, lateral root number, and root dry weight. Shoot dry weight for each plant were also measured. Gene expressions of *ZmZIP1 - 8* in the roots of Wu312 and Ye478 in each single compartment were analyzed. Three technical replicates were performed for each biological replicate.

### Measurement of Physiological Traits

In Experiment 1 and 2, shoots and roots for plants were separately collected in an envelope. All samples were heat-treated at 105°C for 30 min and dried at 75°C until constant weight. Zn concentrations in shoots and roots were analyzed by Inductively Coupled Plasma-Atomic Emission Spectroscopy (ICP-AES). Zn uptake efficiency, Zn transport efficiency, and Zn use efficiency were calculated using the following equations from (1) to (3), respectively.

1. Zn uptake efficiency (μg root dry weight g^−1^) = $\frac{Total\;Zn\;content}{Root\;dry\;weight}$
2. Zn transport efficiency (%) = $\frac{Shoot\;Zn\;content}{Total\;Zn\;content}$
3. Zn use efficiency (μg g^−1^) = $\frac{Total\;dry\;weight}{Total\;Zn\;content}$

In Experiment 3, roots in each single compartment were scanned using Epson scanner and then several root morphological traits of each sample were measured using WinRhizo (Regent Instrument Inc., Quebec, QC, Canada).

### Gene Expression Analysis of *ZmZIP1–8* Genes

Total RNA was isolated from each root sample for Wu312 and Ye478 with TRIzol (Takara). We used 1.5 μg of total RNA to synthesize cDNA. Quantitative real-time polymerase chain reaction (PCR) was performed using SYBR Green Real-time RT-PCR (Applied Biosystems) and an ABI7500 Fast Real-Time PCR System (Applied Biosystems). The primers used for real-time PCR are shown in Supplementary Table 1.

### Statistical Analysis

Each experiment was arranged in accordance with a randomized complete design. Means among different treatments were compared using the least significant difference (LSD) test. Means between inbred lines Wu312 and Ye478 were compared using *t*-test. A probability level of *p* < 0.05 was required for statistical significance.

## Results

### Effects of Different Zn Supplies and Different Stress Time Length on Phenotype, Leaf SPAD and Biomass in Maize

**Figure 2:**
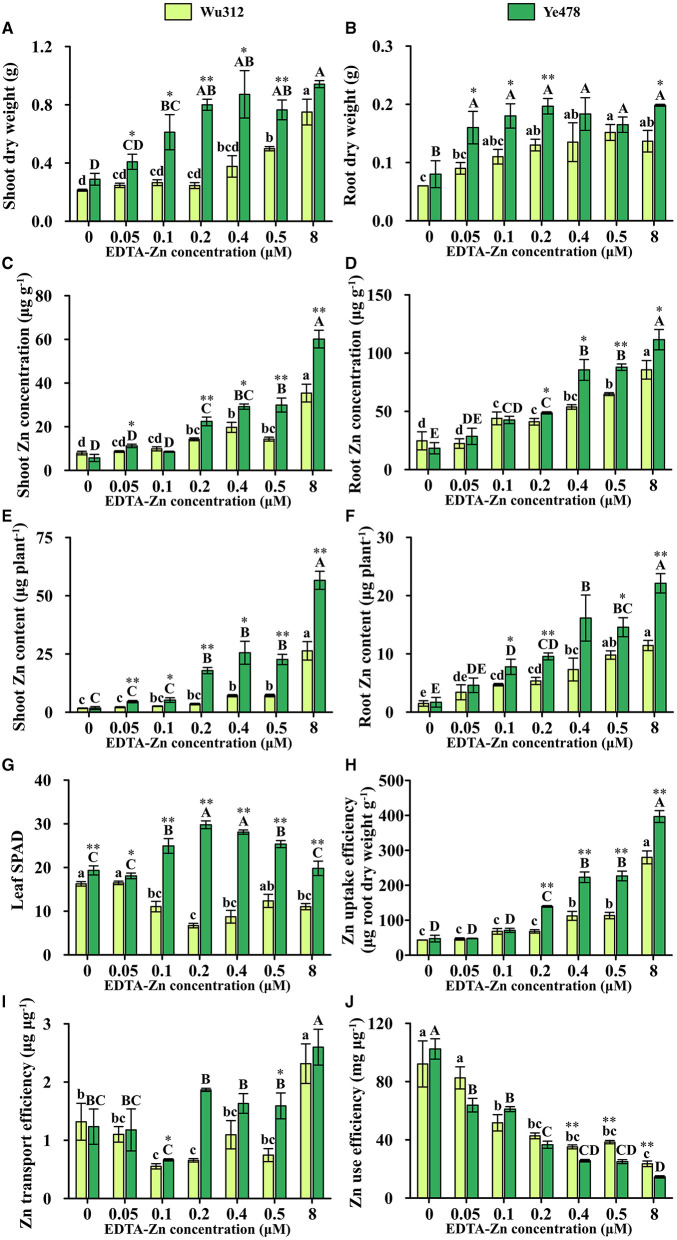
Shoot **(A)** and root **(B)** dry weight, shoot **(C)** and root **(D)** Zn concentration, shoot **(E)** and root **(F)** Zn content, leaf SPAD **(G)**, Zn uptake efficiency **(H)**, Zn transport efficiency **(I)** and Zn use efficiency **(J)** of Zn-sensitive inbred line Wu312 and Zn-tolerant inbred line Ye478 in Experiment 1. Different lowercase and upper letters indicate significant difference (*p* < 0.05) of Wu312 and Ye478 among treatments, respectively. * and ** indicate significant difference between Wu312 and Ye478 at *p* < 0.05 and *p* < 0.01, respectively.

In Experiment 1, when the Zn supply concentration was 0.5 μM, shoot and root dry weights of Ye478 were 154 and 109% higher than those of Wu312, respectively. Compared with Zn-sufficient supply, shoot dry weights of Wu312 and Ye478 under Zn deficient condition (0.5 μM) were decreased by 33.6 and 18.8%, respectively (Figure 2A), while root dry weights were decreased by −11.0 and 16.8%, respectively (Figure 2B).

**Figure 3:**
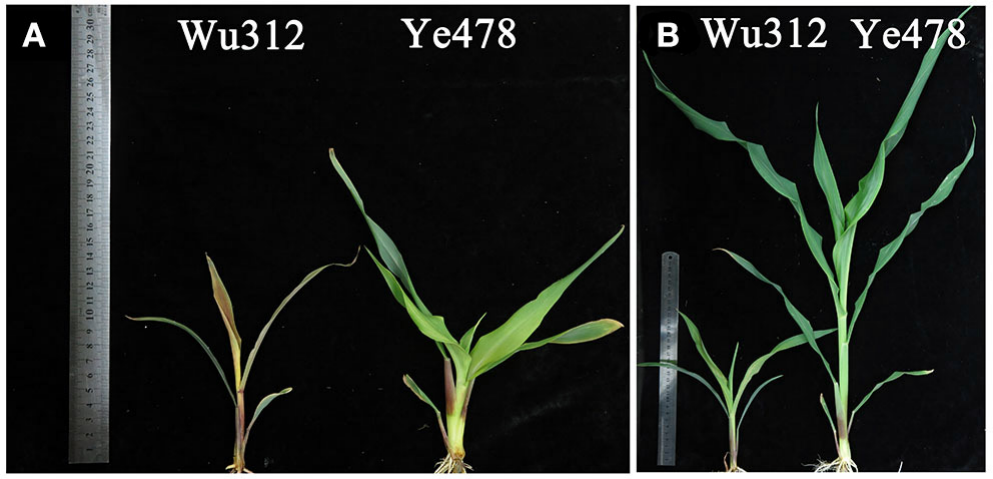
Shoots of Zn-sensitive inbred line Wu312 (left) and Zn-tolerant inbred line Ye478 (right) under different Zn-deficient status at the 21th day after transfer in Experiment 2. **(A)** 0 μM EDTA-Zn; **(B)** 0.5 μM EDTA-Zn. A 30 cm-length ruler was shown in both **(A,B)**.

In Experiment 2, at the nil Zn supply (0 μM), plant of Wu312 was tended to be dead and Ye478 showed serious Zn-deficient symptoms (Figure 3A). When the Zn supply level was increased to 0.5 μM, there were significantly phenotypic difference between them. Few Zn-deficient symptoms were observed in Ye478 (Figure 3B). However, plant growth of Wu312 was strongly depressed, resulting in a shortened and deformed plant and chlorosis with small leaves in the shoots (Figure 3B). When the Zn concentration was 8 μM, Wu312 and Ye478 grew normally (Supplementary Figure 1).

**Figure 4:**
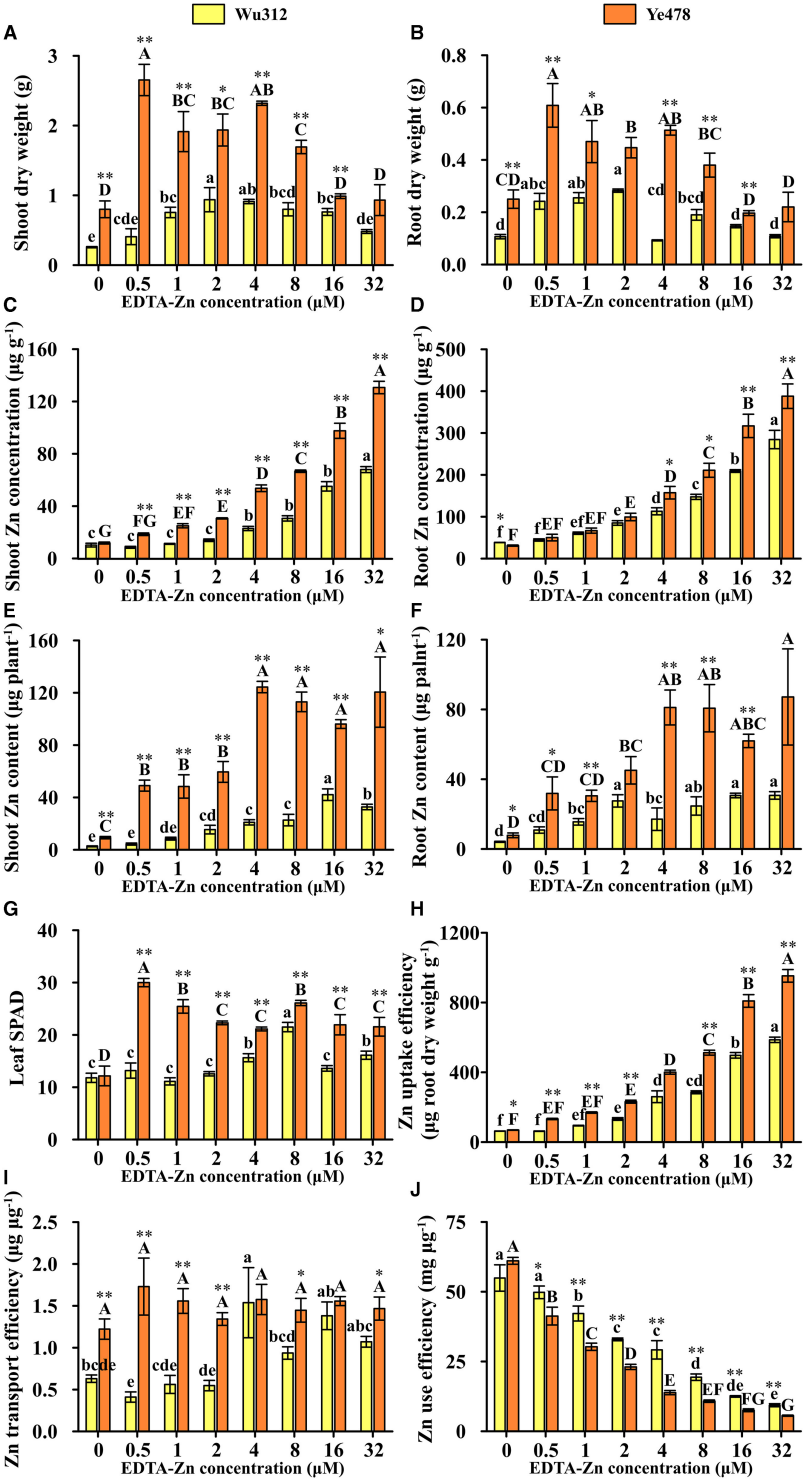
Shoot **(A)** and root **(B)** dry weight, shoot **(C)**, and root **(D)** Zn concentration, shoot **(E)**, and root **(F)** Zn content, leaf SPAD **(G)**, Zn uptake efficiency **(H)**, Zn transport efficiency **(I)**, and Zn use efficiency **(J)** of Zn-sensitive inbred line Wu312 and Zn-tolerant inbred line Ye478 in Experiment 1. Different lowercase and upper letters indicate significant difference (*p* < 0.05) of Wu312 and Ye478 among treatments, respectively. * and ** indicate significant difference between Wu312 and Ye478 at *p* < 0.05 and *p* < 0.01, respectively.

In Experiment 2, when the Zn supply level was 0.5 μM, compared with Zn-sufficient treatment (8 μM), leaf soil plant analysis development (SPAD) of Wu312 was decreased by 38.7% and SPAD of Ye478 was increased by 14.9%. When the Zn supply concentration was increased to 16 and 32 μM, leaf SPAD values of two inbred lines were significantly reduced (Figure 4G). Under different Zn supply concentrations, shoot and root dry weights of Ye478 were significantly higher than those of Wu312 (Figures 4A,B). When Zn concentration, supplied with EDTA-Zn were increased to 16 and 32 μM, there were higher shoot and root Zn concentrations in these two inbred lines (Figures 4C,D), and plant growth was depressed, especially for Ye478. In addition, compared with 8 μM, shoot dry weight of Wu312 under Zn-deficient condition (0.5 μM) was decreased by 49.1%, while shoot and root dry weights of Ye478 were increased by 56.7 and 60.1%, respectively (Figures 4A,B). When supplied with 0.5 μM Zn, the root to shoot ratio of Wu312 was significantly higher than that of Ye478 (Supplementary Figure 2).

Based on visible phenotypic variation of plants, shoot and root dry weight, and leaf SPAD of Wu312 and Ye478 in Experiments 1 and 2, Zn nutritional status of 8 μM was determined to be the Zn-sufficient condition (control treatment). When Zn concentration was 0.5 μM, compared with 21-day Experiment 2, a 14-day Experiment 1 was designed as a short-term zinc deficiency experiment. In addition, shoot dry weights of Ye478 under Zn-deficient (0.5 μM EDTA-Zn), and Zn-sufficient condition (8 μM EDTA-Zn) under short-term stress were 1.5- and 1.3-fold higher than that of Wu312 in Experiment 1, respectively. Under longer-term Zn deficiency stress in Experiment 2, these multiple values were reduced to 6.5 and 2.1, respectively.

**Figure 5:**
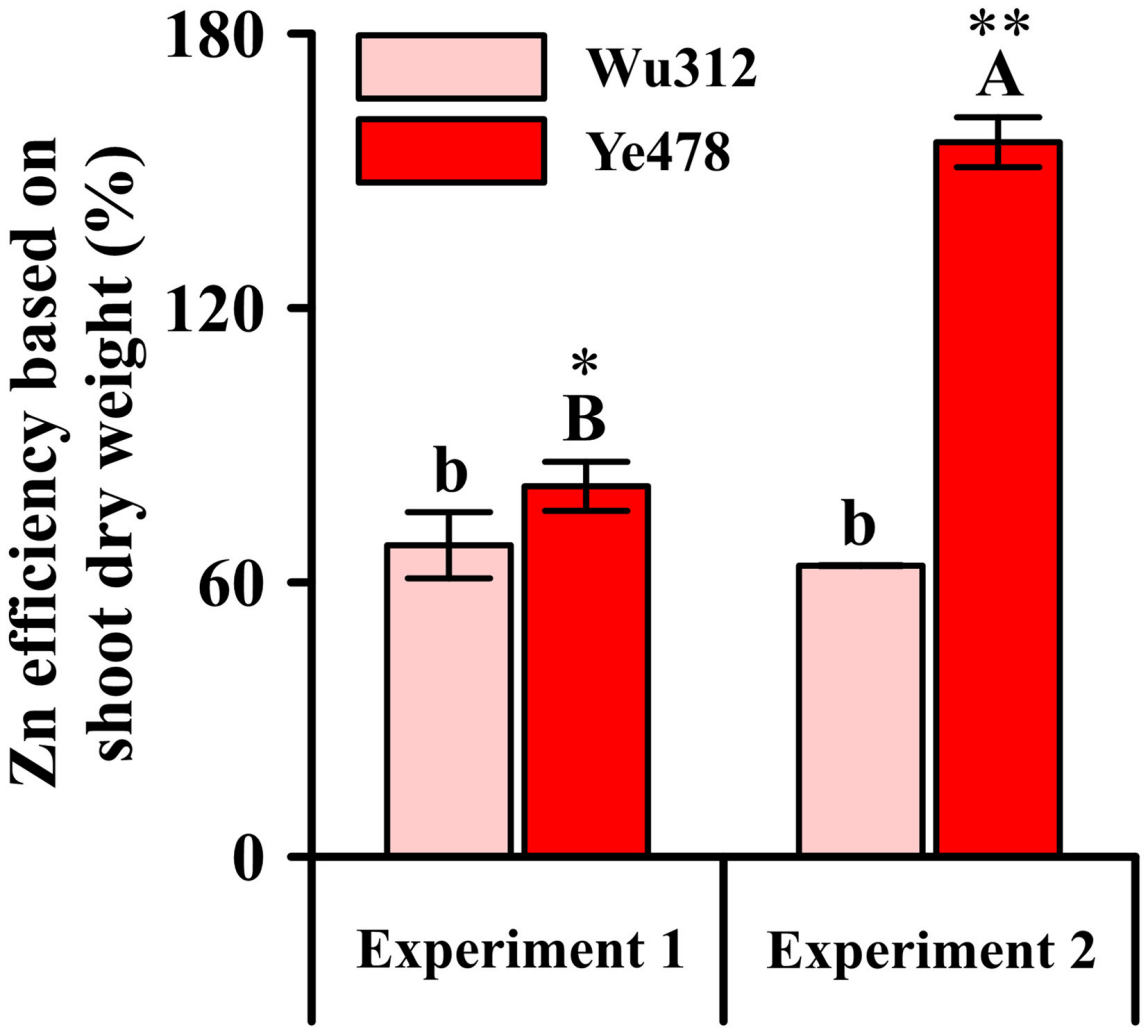
Zn efficiency based on shoot dry weight (%) of Zn-sensitive inbred line Wu312 and Zn-tolerant inbred line Ye478 in Experiment 1 and Experiment 2. Different lowercase and upper letters indicate significant difference (*p* < 0.05) of Wu312 and Ye478 among treatments, respectively. * and ** indicate significant difference between Wu312 and Ye478 at *p* < 0.05 and *p* < 0.01, respectively.

Relative values of shoot dry weight, which were estimated by the ratio of shoot dry weight under 0.5 μM low Zn stress to the shoot dry weight under Zn-sufficient condition (8 μM EDTA-Zn), were considered as Zn efficiency (ZE) based on shoot dry weight. ZEs for Zn-sensitive inbred line Wu312 showed no significant difference between Experiment 1 and 2. However, ZE for Zn-tolerant inbred line Ye478 in Experiment 2 was 192.9% higher than that in Experiment 1 (Figure 5). This finding showed that Zn-efficient inbred line Ye478 was able to utilize limited Zn to maintain plant growth, while Zn-inefficient Wu312 was depressed to accumulate enough shoot biomass in response to long-term Zn deficiency. Our results further confirmed that longer term Zn starvation not only led to a great reduction in ZE of Zn-sensitive genotype and a significant increase in ZE of Zn-tolerant genotype, but also enlarged the difference in ZE between Zn-sensitive and Zn-tolerant genotypes in maize.

### Effects of Different Zn Supplies and Different Stress Time Length on the Zn Concentration, Zn Content and Zn Uptake, Transport, and Use Efficiency in Maize

In Experiments 1 and 2, shoot and root Zn concentration of Wu312 and Ye478 gradually enhanced with the increase of Zn supply (Figures 2C,D, 4C,D). Except for the nil Zn supply, the shoot Zn concentration of Ye478 was significantly higher than that of Wu312. In Experiment 1, compared with sufficient Zn condition (0.8 μM), low Zn-deficiency stress (0.5 μM EDTA-Zn) decreased Zn concentrations in the shoots and roots of Wu312 and Ye478 by 59.5 and 24.3%, and 50.2 and 21.2%, respectively (Figures 2C,D). In Experiment 2, low Zn stress (0.5 μM) decreased shoot and root Zn concentration of Zn-sensitive inbred line Wu312 by 72.1 and 69.9%, respectively. Shoot and root Zn concentration of Zn-tolerant inbred line Ye478 was 72.2 and 76.2% decreased by 0.5 μM Zn-stress deficiency, respectively (Figures 4C,D). This indicated that compared with short-term Zn deficiency, deficiency in Zn decreased Zn concentration in maize more under longer-term low-Zn stress. In addition, Zn concentration decreased more significantly in roots than in shoots under longer Zn deficiency. However, Zn content mostly decreased more in shoots than in roots when the Zn starvation time was longer (Figures 4E,F).

In Experiment 1, compared with Zn sufficient condition (8 μM), Zn uptake and transport efficiency of Wu312 and Ye478 under Zn deficient condition (0.5 μM) was decreased by 59.5 and 67.8%, and 42.9 and 38.8%, respectively (Figures 2H,I). And Zn use efficiency was increased by 63.2 and 71.8%, respectively (Figure 2J). In Experiment 2, Zn uptake and transport efficiencies of Wu312 and Ye478 were reduced by 78.1 and 56.1%, and 74.2 and −19.6%, respectively, and Zn use efficiencies of Wu312 and Ye478 were increased by 156.9 and 282.4%, respectively. In Experiment 2, Zn uptake efficiency of Ye478 was significantly higher than that of Wu312 except for the nil Zn supply. Zn uptake efficiency of Wu312, Ye478, and the difference between them enhanced with the increase of Zn supply level (Figure 4H). Transport efficiency of Wu312 was higher than Ye478 in all treatments (Figure 4I). Zn use efficiency of two inbred lines gradually decreased with EDTA-Zn concentration. Except for the treatment without Zn supply, Zn use efficiency of Wu312 was also higher than that of Ye478 (Figure 4J). These findings showed that Zn uptake efficiency of maize plants decreased more, and Zn use efficiency increased more under longer-term Zn-deficiency stress.

### Effects of Heterogeneous Zn Supply on Root Morphology and Biomass in Maize

**Figure 6:**
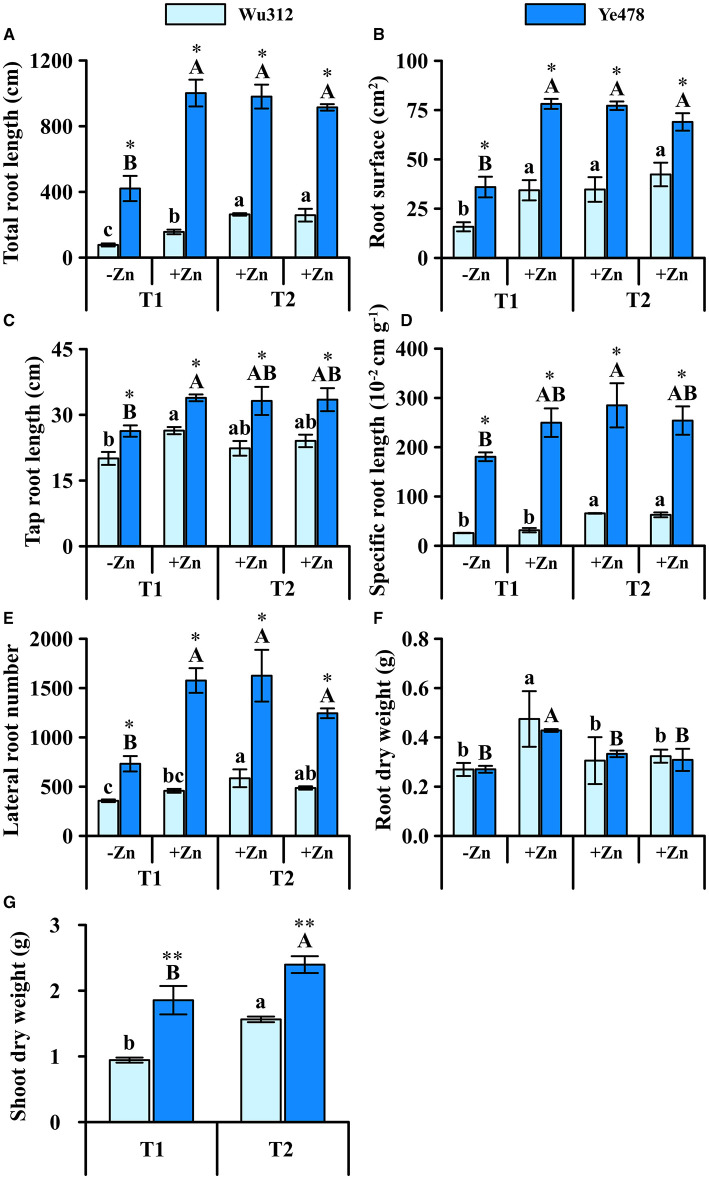
Total root length **(A)**, root surface **(B)**, tap root length **(C)**, specific root length **(D)**, lateral root number **(E)**, root **(F)**, and shoot **(G)** dry weight of Zn-sensitive inbred line Wu312 and Zn-tolerant inbred line Ye478 under split-supply with –Zn (0.5 μM) and +Zn (8 μM) (T1) and with +Zn (8 μM) and +Zn (8 μM) (T2) in Experiment 3. Different lowercase and upper letters indicate significant difference (*p* < 0.05) of Wu312 and Ye478 among treatments, respectively. * and ** indicate significant difference between Wu312 and Ye478 at *p* < 0.05 and *p* < 0.01, respectively.

Under the condition of heterogeneous Zn supply, total root length, root surface area, and lateral root number of the Wu312 and Ye478 under Zn deficiency were significantly reduced (Figures 6A,B,E) compared to the homogeneous Zn supply. In the +Zn regions of T1 and T2, there was no significant difference in these three traits (total root length, root surface area, and lateral root number) of Ye478. On the other hand, the total root length of Wu312 was significantly reduced by Zn deficiency, while the number of lateral roots also decreased (Figure 6E).

Specific root length of two inbred lines differed greatly, but the difference in main root length was relatively small (Figure 6D). In the +Zn region, taproots of two inbred lines were longer than those in the -Zn region (Figure 6C). Under heterogeneous Zn supply (T1), Ye478 and Wu312 produced more root biomass (0.74 and 0.70 g) in the -Zn region than in the +Zn region (0.63 and 0.64 g) (Figure 6F). In addition, Wu312 accumulated the higher root dry weight in the +Zn regions of T1 and T2 (Figure 6F). However, shoot dry weights of Wu312 and Ye478 (0.94 and 1.85 g) were still lower in the T1 (1.56 g) than in the T2 (2.39 g). The difference between Wu312 and Ye478 was greater in T1 (1.97 times) than in T2 (1.53 times) (Figure 6G). These implicate that there are significant genotypic differences in response to heterogeneous Zn.

### The Effect of Heterogeneous Zn Supply on the Expression of *ZmZIP*s

**Figure 7:**
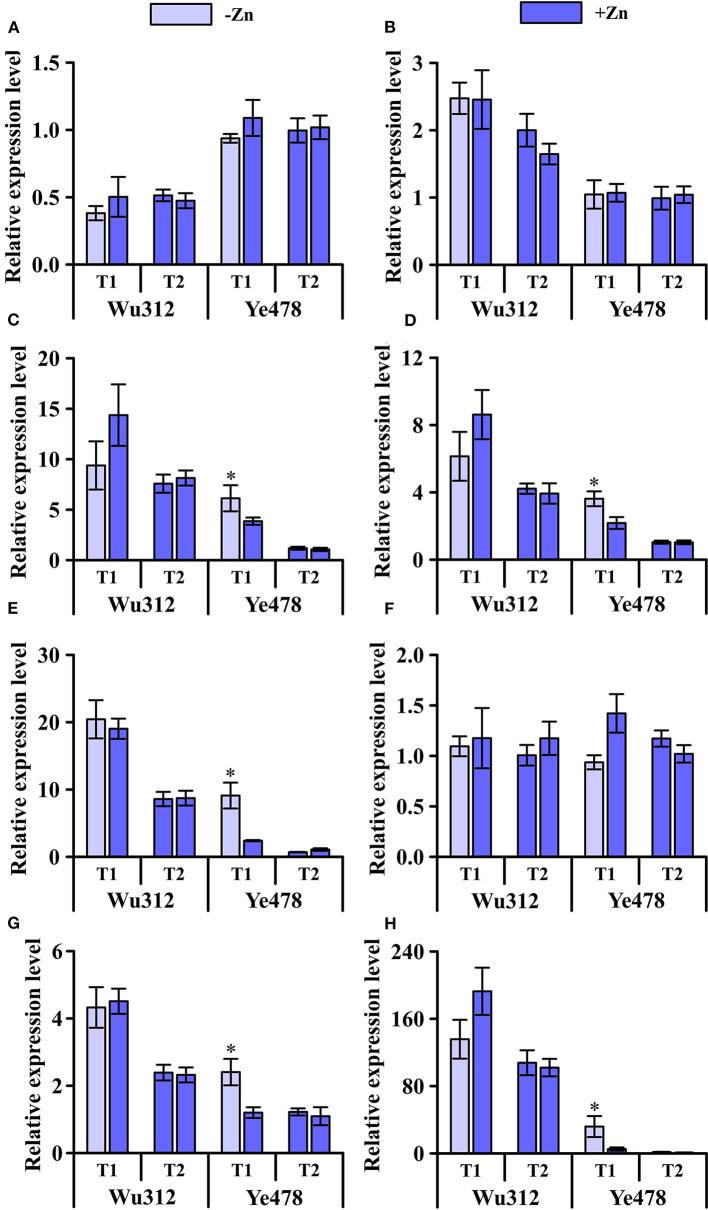
Expression of *ZmZIP1* **(A)**, *ZmZIP2* **(B)**, *ZmZIP3* **(C)**, *ZmZIP4* **(D)**, *ZmZIP5* **(E)**, *ZmZIP6* **(F)**, *ZmZIP7* **(G)**, and *ZmZIP8* **(H)** genes in the roots of Zn-sensitive inbred line Wu312 and Zn-tolerant inbred line Ye478 under split-supply with –Zn (0.5 μM) and +Zn (8 μM) (T1 treatment) and with +Zn (8 μM) and +Zn (8 μM) (T2 treatment) in Experiment 3. *Indicate significant difference between the –Zn (+Zn) region and +Zn (+Zn) region in T1 (T2) treatment at *p* < 0.05.

Under the condition of heterogeneous Zn supply, compared with homogeneous Zn-sufficient supply, the expression levels of *ZmZIP1, ZmZIP2*, and *ZmZIP6* showed no significant difference (Figure 7). However, the expression levels of *ZmZIP1* and *ZmZIP2* in roots of different Zn efficiency genotypes were different (Figures 7A,B) while *ZmZIP6* showed no significant difference (Figure 7F). Uneven supply of Zn induced an increase in the expression of *ZmZIP3* and *ZmZIP4* in the roots of Wu312 and Ye478, while the expression in the +Zn region was higher in the root of Wu312 (Figures 7C,D). Heterogeneous Zn supply induced higher expression of both *ZmZIP5* and *ZmZIP7* in the two inbred lines. There was no significant difference in the expression levels of *ZmZIP5* and *ZmZIP7* for Wu312 between the –Zn and +Zn regions of T1 while Ye478 showed a significant increase in the -Zn region (Figures 7E,G). Compared with T2, the expression of *ZmZIP8* was higher in T1, and the expression level of *ZmZIP8* was higher than that of *ZmZIP1-7* (Figure 7H). These results showed that compared with split-supply with sufficient Zn (+Zn/+Zn), *ZmZIP*s expressed differently in roots under split-supply with deficient and sufficient Zn (–Zn/+Zn). Also, the expression levels of these genes in Zn-efficient and Zn-inefficient genotypes were also different.

## Discussion

### Differences in Physiological Responses of Seedling Maize to Different Zn Stress Levels and Stress Time Lengths

In Experiment 1, shoot dry weight of Ye478 under Zn-deficient condition (0.5 μM) was 1.5 times higher than that of Wu312 despite there being no significant difference in root dry weight between two inbred lines (Figures 2A,B). However, in our previous results (unpublished data), Zn-inefficient inbred line, K22, was selected from 513 core lines (excluding Wu312) worldwide. After a 14-day Zn deficiency, K22 displayed obvious Zn-deficient symptoms which was also observed in Wu312. But these symptoms as well as the phenotypic difference between Zn-deficient and Zn-sufficient plants of K22 gradually decreased when Zn deficiency stress duration was extended to 21 days (Supplementary Figure 3). Unlike K22, our results in this study showed that longer-term stress not only led to decrease in the ZE of Wu312 but also enlarged the difference between Wu312 and Ye478 (Figure 5). Hence, a 21-day Zn deficiency experiment was designed to analyze the difference in Zn deficiency tolerance between Zn-sensitive and Zn-tolerant inbred lines.

Apart from extending stress duration, we also investigated the effects of excess Zn on maize via increasing the Zn supply to 16 and 32 μM. Previous studies have shown that excess Zn inhibited the production of biomass, chlorophyll, total soluble protein, and strongly increased accumulation of Zn in both root and shoot mainly because Zn-induced oxidative stress can cause loss of plasma membrane integrity, electrolyte leakage, increase in malondialdehyde content, and damage photosynthesis to produce reactive oxygen species (Cambrollé et al., 2012; Islam et al., 2014; Anwaar et al., 2015).

In addition, the results from our previous experiments (unpublished) indicated that the root surface of Wu312 have begun to rot and become grayish brown since the 21st day after transfer, which would probably lead to necrosis in the whole plant (Supplementary Figure 3). But the roots of K22 stayed healthy without necrosis, which was consistent with the phenotypes in the roots of Ye478 (Supplementary Figure 3). These suggest that roots play important roles in the tolerance to Zn deficiency at maize seedling stage. Therefore, Experiment 3 was designed to investigate the responses of root morphology and the genes relevant with Zn-deficiency tolerance to low Zn stress by the means of simulating heterogeneous Zn supply in the split-root experiments.

The R/S ratio of genotypes increased as Zn application decreased (Sadeghzadeh et al., 2009). The two inbred lines in this study also showed similar trends. Some studies have concluded that genotypes with higher Zn efficiency have higher R/S ratio (Rengel and Romheld, 2000; Cakmak and Braun, 2001) but compared with Ye478, Wu312 had a similar or higher R/S ratio at all Zn supply levels (Supplementary Figure 2). Cakmak et al. (1996a) described the increased root growth under Zn deficiency as a result of Zn-deficiency induced photo-oxidative damage in shoots, which caused lower shoot growth. Therefore, this study also suggests that R/S ratio is not an appropriate index for evaluating the ability to withstand low Zn stress.

As the level of Zn supply kept increasing, the shoot Zn concentration, root Zn concentration, and Zn uptake efficiency all increased. However, the biomass did not show a trend of increasing (Figures 2, 4). According to the results of shoot Zn concentration and shoot dry weight in Experiment 2, there was no influence of Zn nutrient dilution effect. This indicated that although maize absorbed more Zn nutrients, these nutrients were not effectively transported and utilized. These nutrients neither produced more biomass (Figures 2A,B, 4A,B) nor increased chlorophyll (Figures 2G, 4G). In other words, simply providing more Zn in the medium does not necessarily promote plant growth.

Comparing the results of dry matter weight, SPAD value, and phenotype observation in Experiment 1 and Experiment 2, when the Zn supply level was between 0.2 and 0.5 μM, the growth difference between the two genotypes was the most significant. Compared with the Zn-sufficient condition (8 μM), Ye478 could continue to grow healthily, while the development of Wu312 was significantly inhibited. This may be because genotypes with different tolerance to Zn stress have different minimum Zn requirements (Grewal and Williams, 1999). The internal mechanism for the differences may be that the Zn-efficient genotype maintains higher cytoplasmic Zn concentrations for biochemical processes (Hacisalihoglu et al., 2004). There are also some physiological processes that may be related to it, such as root growth, phloem mobility, tolerance of radical oxygen stress, proton exudation, mycorrhizal colonization, efflux of phytosiderophores and low molecular weight organic acids, and formation of iron plaques on roots (Impa et al., 2013a,b; Rose et al., 2013). In many studies in wheat and bean, it has been found that shoot Zn concentration is not a reliable parameter for screening genotypes with high Zn efficiency and low Zn efficiency (Rengel and Graham, 1995; Cakmak et al., 1999; Torun et al., 2000; Hacisalihoglu et al., 2001). Despite this, Sadeghzadeh et al. (2009) suggested that shoot Zn concentration can be used in assessment of barley genotypes and may be useful criteria in screening large genotypes aimed at developing molecular markers for Zn efficiency. The results of this study also showed that the shoot Zn concentration may be an important parameter for screening genotypes in maize. This needs to be verified in more maize materials. Genc et al. (2006) proposed that visual symptoms of the severity of Zn deficiency was a good predictor of Zn efficiency. In summary, combined with the results of this study and previous studies, dry weight, visual evaluation of Zn deficiency symptoms, SPAD value, and Zn content are effective parameters for evaluating the difference in Zn efficiency of different genotypes.

To date, few studies have reported the effect of different Zn-deficiency stress time length on physiological traits of Zn-sensitive and Zn-tolerant genotypes in maize despite several reports in other crops, such as wheat (Rengel and Graham, 1995, 1996; Cakmak et al., 1996a), barley (Tiong et al., 2015), and rice (Impa et al., 2013b). Consistent with previous results, when the stress time length was extended, Zn-tolerant genotype kept stable shoot growth and Zn-sensitive genotype showed great reduction in shoot dry weight which, therefore, also increased the differences of biomass and relative values of biomass between them (Rengel and Graham, 1996; Impa et al., 2013b). ZE is obviously reduced by extended time length of Zn deficiency as reported by previous studies (Rengel and Graham, 1996; Impa et al., 2013b). This was observed in Zn-sensitive genotype but not in Zn-tolerant genotype in our results. Beyond that, Zn uptake was also strongly reduced by long-term Zn starvation, but Zn-tolerant genotypes maintained more efficient uptake compared with Zn-sensitive genotypes, which was found in our results and other research (Rengel and Graham, 1996). It is reported that phytosiderphore release rate is significantly enhanced, and that the difference between Zn-efficient and Zn-inefficient genotypes is enlarged by long-term low zinc stress in wheat (Cakmak et al., 1996b). These findings demonstrate that increased synthesis and release of phytosiderophores is involved in zinc deficiency tolerance mechanism via affecting mobilization of zinc from soluble zinc pools and adsorption sites within the root rhizosphere and plants (Cakmak et al., 1996b).

Despite the physiological reasons for the differences in Zn efficiency of different genotypes being previously analyzed in many studies, there is still no clear and systematic explanation for mechanism underlying the tolerance to Zn deficiency. In this study, when the Zn supply level was ≥0.2 μM, the Zn uptake and transport efficiency of Ye478 were significantly higher than that of Wu312. Because the morphological response of the maize root system is important for absorption, *ZIP* genes play important roles in enhancing the Zn uptake efficiency and transport of Zn under low Zn stress. Therefore, this study analyzed whether root variation and *ZmZIP*s are important reasons for differences in Zn efficiency by simulating the heterogeneity of soil Zn nutrition.

### Root Morphological Plasticity of Maize Inbred Lines With Different Zn Efficiency in Response to Heterogeneous Zn Supply

Plants can develop root systems with morphological plasticity to forage nutrients distributed heterogeneously in soils (Liu et al., 2020). Until now, little is known about how the root of maize responds to heterogeneous Zn supply. So, when characterizing the root morphological and physiological adaptations to Zn-rich and Zn-poor patches of maize, we can only refer to the results of research on the response of the root system to the heterogeneity of nutrients such as nitrogen, phosphorus, and potassium. In addition, some nutrient-responsive genes regulating root development respond to more than one nutrient deficiency (Giehl et al., 2014).

Under the condition of non-uniform Zn supply, the root development of the two inbred lines in the Zn-stressed area was significantly weaker than that in the +Zn area and the control (Figure 6). This shows that maize can coordinate the distribution of assimilate carbon to the root system in the nutrient-rich area according to the environmental characteristics and use the changes of physiological morphology to improve the absorption of nutrients by the root system. They both displayed carbon saving strategies in response to heterogeneous Zn supply (Yu et al., 2014a). Due to the limited nutrient supply, the growth of roots in low-Zn areas is restricted to minimize the local metabolic cost (Fisher et al., 2002). Studies have shown that increased root growth in one patch can cause reduced growth outside the patch (Linkohr et al., 2002), suggesting carbon limitation (Irving et al., 2019). The results of this study are similar to the root development observed by Giehl et al. (2012) under heterogeneous iron supply. Local iron supply restores root growth only in those lateral roots that have access to iron and induce AUX1 to promote auxin accumulation (Giehl et al., 2012; Yu et al., 2014b).

The ability to forage for localized supplies of nutrients is important for capturing sufficient amounts of limiting nutrients and to compete for limiting resources (Jansen et al., 2006). Root foraging and competition for heterogeneous nutrients have nutrient specificity and genotypic differences (Farley and Fitter, 1999; Li et al., 2014). Compared with Ye478, the root morphology of Wu312 was more sensitive to the response of non-uniform Zn supply (Figure 6). Compared with the control, the root growth of Ye478 in the +Zn region had no significant difference; meanwhile several root traits of Wu312 were lower than the control in the +Zn region. And compared with the control, the heterogeneous Zn supply reduced the shoot dry weight of Wu312 by 39.7%, while Ye478 was reduced by 22.6% (Figure 6G). Although Wu312 was quite different from Ye478 in other root traits under heterogeneous nutrient conditions, the difference between the two genotypes was small in terms of tap root length. The response of Wu312 may be similar to strategy I genotype (Col-0) in dealing with potassium deficiency in *Arabidopsis*. Col-0 maintains the elongation of main root but compromises lateral root growth (Kellermeier et al., 2013). This may be mainly because the genomic hubs exist in the coordinated control of root growth under stress conditions.

Due to nutrient stress, plants can increase resource acquisition by increasing their R/S ratio (Hermans et al., 2006). Both inbred lines increased the root dry weight after the Zn deficiency signal was sensed locally. Consequently, the root dry weights of Wu312 and Ye478 were significantly higher under heterogeneous Zn supply than under homogeneous Zn supply. This shows that local Zn deficiency in plant roots may affect plant carbon allocation by coordinating local and systemic signal transduction (Gao et al., 2014; Giehl et al., 2014).

The internal regulatory gene is likely to be similar to NRT1.1 (nitrate transporter/sensor) in *Arabidopsis*. Local nitrate signal is sensed by NRT1.1/NPF6.3 which then triggers the signaling pathway involving the MADS-box transcription factor ANR1 to stimulate root growth in nitrate-rich patches (Bouguyon et al., 2015; Liu et al., 2020). Meanwhile, NRT1.1 acts locally to modulate both auxin level and meristematic activity for inhibiting the growth of lateral roots at low nitrate (Mounier et al., 2014). Although there was no significant difference in single root dry weight under heterogeneous Zn supply between Wu312 and Ye478, the degree of inhibition on the shoot dry weight of Wu312 was still more than that of Ye478. This may be due to the differences in the Zn uptake efficiency and transport efficiency of the roots of Wu312 (Figures 2H,I, 4H,I).

### Non-uniform Zn Supply Induces Differences in the Expression of *ZmZIP*s

The *ZIP* genes are crucial in plant adaptation to low and fluctuating Zn availability in soil (Colangelo and Guerinot, 2006). The contribution of ZIPs to Zn absorption and translocation has been studied in *Arabidopsis* and rice. However, studies on the response of *ZIP* genes to Zn deficiency in these crops are mainly limited to a single line. Moreover, the current understanding of the functions of *ZIP* genes in maize is still limited. Only by comparing the homologs closely related to ZmZIP proteins (Li et al., 2013; Mondal et al., 2014; Ajeesh Krishna et al., 2020) can we understand the related functions of the *ZIP* family genes in maize.

This study also analyzed the expression level of *ZIP*s that were not treated with non-uniform Zn supply under the same Zn supply conditions (unpublished). The expression levels of each gene in the two lines are basically consistent with the results of this study. According to the results of this study, the expression of *ZmZIP1* in two inbred lines under the condition of non-uniform Zn supply did not show a significant difference compared with the control (Figure 7A). The molecular mechanism of members of the ZIP family was less understood. OsZIP1 has long been considered to account for the Zn uptake, but recently, it has been confirmed to be a metal detoxified transporter in both endoplasmic reticulum and plasma membrane, rather than for the uptake of Zn (Ramesh et al., 2003; Liu et al., 2019). In addition, *OsZIP1* is abundantly expressed in roots throughout the life span (Liu et al., 2019). There was also no significant difference in the expression of *ZmZIP2* in Ye478 and Wu312 between non-uniform Zn supply and uniform Zn supply. But unlike *ZmZIP1*, the expression level of *ZmZIP2* in Wu312 was higher than that of Ye478 (Figure 7B). Northern blot analysis demonstrated that *ZIP2* is not Zn responsive (Grotz et al., 1998). The transcription factors bZIP19 and bZIP23 were reported to control the expression of many Zn-deficiency genes. However, expression of *ZIP2*, which has no Zn deficiency response element sequence in its promoter, is not induced when comparing *m19m23* double mutant and wild type (Assunção et al., 2010). Therefore, it is speculated that *ZmZIP2* may not contribute to the tolerance to low Zn stress.

Compared with the control, non-uniform Zn supply increased the expression of *ZmZIP3* in Ye478 and Wu312 (Figure 7C). Among them, under the condition of non-uniform Zn supply, the expression level of *ZmZIP3* in Ye478 root under low Zn stress was significantly higher than that in other treatments. ZmZIP3 is functional Zn transporter of leaf-specific and is shown to be localized to the plasma membrane and endoplasmic reticulum. Overexpressing *ZmZIP3* represses Zn accumulation in the shoot of transgenic plants while that in the root is enhanced (Li et al., 2015). However, neither *OsZIP3* gene expression nor encoded protein was affected by either deficiency or toxic levels of Zn. OsZIP3 is localized to xylem transfer cells in enlarged vascular bundles (EVBs) of the nodes and responsible for the preferential distribution of Zn to developing tissues (Sasaki et al., 2015). ZmZIP4 was significantly increased under non-uniform Zn supply compared with control treatment, especially in the –Zn region. However, the expression of ZmZIP4 in Wu312 was not significantly increased in the –Zn region compared with the +Zn regions (Figure 7D). *OsZIP4*, which encodes functional transporter of Zn, is highly expressed under conditions of Zn deficiency in roots and shoots of rice, especially in phloem cells and the meristem (Ishimaru et al., 2005). Transgenic rice plants (overexpressed *OsZIP4* gene) mainly accumulate Zn in the roots rather than in shoots. OsZIP4 is a Zn transporter that localizes to the phloem cells of stems, vascular bundles of leaves, and roots. Constitutive expression of it changes the Zn distribution within rice plants (Ishimaru et al., 2007).

Under the condition of non-uniform Zn supply, low Zn stress induced the expression level of *ZmZIP5* in the two lines to be significantly higher than the control. The expression of ZmZIP5 in Ye478 roots was significantly increased in the –Zn region compared with the +Zn regions (Figure 7E). At the seedling stage, high levels of Zn are found in the roots and shoots of Ubi-*ZmZIP5* plants (*ZmZIP5* is constitutively overexpressed). However, low levels are found in the *ZmZIP5*i (RNAi line) plants. *ZmZIP5* may play a key role in Zn uptake and root-to-shoot translocation (Li et al., 2019). When *ZmZIP5* is expressed in yeast cells, it can reverse the growth defects of Zn and Fe-uptake-deficient double mutants (Li et al., 2013). The cDNA from *OsZIP5*, which is specific to Zn, complements the growth defect of a yeast Zn-uptake mutant (Lee et al., 2010). Overexpression of *OsZIP5* results in elevated Zn levels in rice roots but caused it to decrease in the shoots (Li et al., 2019).

The ZmZIP7 has been proven to be a functional Zn transporter. In this study, compared with the control, when the root system received the signal of Zn deficiency, the expression of *ZmZIP7* was increased (Figure 7G). Li et al. (2016) detected the expression profiles of *ZmZIP7* in maize and found high levels in roots, stems, leaves, and seeds. Zn concentrations were elevated in the transgenic *Arabidopsis* overexpressing *ZmZIP7* plants (Li et al., 2016). The main reason is that ectopic overexpression of *ZmZIP7* may enhance the transcription levels of key genes responsible for Zn uptake, transport, and storage. *HvZIP7*, primarily in epidermal cells and vascular tissues of roots, is highly induced in both roots and shoots by Zn deficiency, and the protein is localized in the plasma membrane. According to the functions of HvZIP7 and its close homolog OsZIP7 (implicate in xylem loading of Zn), ZmZIP7 may display characteristics of low-affinity Zn transport in the plant (Tiong et al., 2014).

The *ZmZIP8* has similar expression characteristic with the two homologous genes *OsZIP8* and *OsZIP9*. The results of this study showed that under the condition of normal Zn supply, the expression of *ZmZIP8* in two lines, particularly Ye478, was inhibited. However, the expression level was increased under the condition of non-uniform Zn supply. Moreover, the expression level of *ZmZIP8* was higher than that of *ZmZIP1-7* (Figure 7H). This is similar to the research results of Yang et al. (2020) who found that *OsZIP9* was expressed mainly under Zn-deficient conditions, and greatly suppressed by high Zn conditions. It showed strong Zn influx transport activity in the epidermal and exodermal cells of lateral roots. OsZIP9 (*K*_m_ value: 22.04 μM) demonstrated a much stronger activity than OsZIP5 (*K*_m_ value: 2.49 μM) and other rice ZIP proteins (Yang et al., 2020). These indicate that ZIP9 may function as a critical regulator maintaining Zn homeostasis and adapting to varying Zn environments by adjusting its expression level based on uptake requirements. In addition, Huang et al. (2020) considered OsZIP9, localized at the root exodermis and endodermis, functioned as a high-affinity influx transporter for Zn and involved in Zn uptake in rice under Zn deficiency. When expressed in yeast cells, OsZIP8 can complement a Zn-uptake-deficient yeast mutant. *OsZIP8* is induced in both rice roots and shoots by Zn deficiency, it may encode a Zn transporter protein in rice (Yang et al., 2009).

In this study, other *ZmZIP*s in the roots of two inbred lines were induced by Zn deficiency with the exception of *ZmZIP1, ZmZIP2*, and *ZmZIP6*. Summarizing the research results of related genes and homologous genes, these isoforms may play different physiological functions. Several genes induced by Zn deficiency in this study are likely to influence each other. The coordinated expression of multiple Zn-inducible *ZmZIP*s may be important for regulating Zn homeostasis in response to low Zn stress. The overlapping expression was observed for three *OsZIP* genes of Zn-inducible *ZIP* genes in epidermal or cortical and stelar cells of rice roots (Bashir et al., 2012).

## Conclusions

In this study, with the extension of Zn deficiency stress duration, ZE based on shoot dry weight for Ye478 was increased by 92.9%, while the ZE for Wu312 displayed no significant changes. This showed that the Zn-efficient genotype could use limited Zn to maintain its growth. However, the Zn-inefficient genotype grew very slowly, and the difference between them would become larger. Visual evaluation of Zn deficiency symptoms, dry weight, SPAD value, and Zn content were effective parameters for evaluating the difference in Zn efficiency of different genotypes. Under the condition of heterogeneous Zn supply, maize at seedling stage promoted the development of roots in local areas with higher Zn concentration and inhibited the growth of some related root parameters in the -Zn region. Compared with homogeneous Zn supply, shoot dry weights of two inbred lines under heterogeneous Zn supply were significantly reduced and their difference became larger. Moreover, under the condition of divided roots, the expression of *ZmZIP*s in different genotypes were different when dealing with non-uniform Zn supply.

## Data Availability Statement

The original contributions presented in the study are included in the article/Supplementary Material, further inquiries can be directed to the corresponding author/s.

## Author Contributions

JX and XW performed the experiments and analyzed the data. JX, XW, and HZ wrote the manuscript. FY designed the study and modified the manuscript. All authors contributed to the article and approved the submitted version.

## Funding

The National Key Research and Development Program of China (2016YFD0200405).

## Conflict of Interest

The authors declare that the research was conducted in the absence of any commercial or financial relationships that could be construed as a potential conflict of interest.

## Publisher's Note

All claims expressed in this article are solely those of the authors and do not necessarily represent those of their affiliated organizations, or those of the publisher, the editors and the reviewers. Any product that may be evaluated in this article, or claim that may be made by its manufacturer, is not guaranteed or endorsed by the publisher.
