# Supplementary material for: Maize Genotypes With Different Zinc Efficiency in Response to Low Zinc Stress and Heterogeneous Zinc Supply
Source: Front Plant Sci. 2021 Oct 8;12:736658. doi: 10.3389/fpls.2021.736658 (PMC8531504; doi:10.3389/fpls.2021.736658)
Supplement: Supplementary file 1 [file Data_Sheet_1.docx]

**Supplementary material**

| **Table S1** The primers used for Real-time PCR | | | |
| --- | --- | --- | --- |
| Gene name | NCBI sequence | Primer | Sequence 5'to 3' |
| *ZmZIP1* | NM_001137726 | ZmZIP1F | CCTCTCTGCGTTGGTTGCTCT |
|  |  | ZmZIP1R | TTGATGGTTGTTTTCTGGTCGT |
| *ZmZIP2* | NM_001159169 | ZmZIP2F | CCACAAATGGCACGAGGTCT |
|  |  | ZmZIP2R | CGAAGACGGAGTGGAAGCAAA |
| *ZmZIP3* | NM_001155536 | ZmZIP3F | GCCTCTTGTTGGTGCCCTTA |
|  |  | ZmZIP3R | TCAACAATGAACGCTGTAGTGCT |
| *ZmZIP4* | HM048832 | ZmZIP4F | CCTTCTTCTCGCTCACCGCT |
|  |  | ZmZIP4R | AGCCTCGGGTTGCTGAAGT |
| *ZmZIP5* | NM_001154257 | ZmZIP5F | GCACATAGGCATAGCCACGC |
|  |  | ZmZIP5R | ACGCCCAAAGATAGCCCGAT |
| *ZmZIP6* | NM_001156151 | ZmZIP6F | ATCAGGTGTTCGAGGGGATG |
|  |  | ZIP6R | TGCTATCGTCGTAGCCAGTC |
| *ZmZIP7* | NM_001157018 | ZmZIP7F | ACTAGGTGGGTGCATTGCTCAG |
|  |  | ZmZIP7R | TGCCAGCAGATACCGAGTCAA |
| *ZmZIP8* | NM_001154769 | ZmZIP8F | CGTGTCATCGCTCAGGTTCTTG |
|  |  | ZmZIP8R | CCCTCGAACATTTGGTGGAAG |
| (Li et al., 2013) | | | |


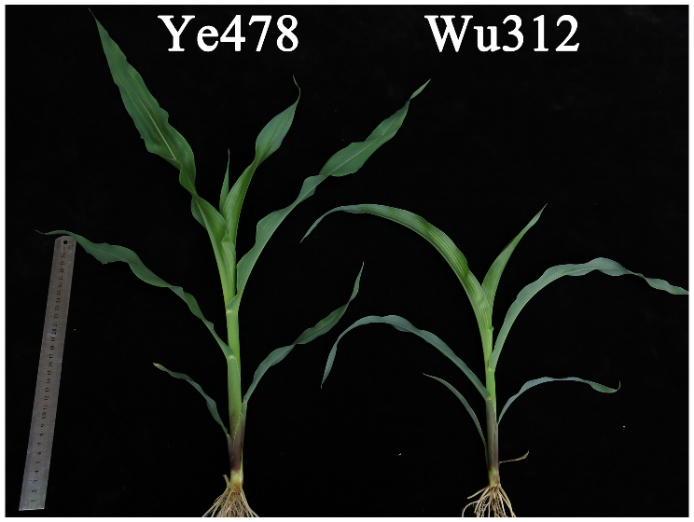


**Fig. S1** Zn-tolerant inbred line Ye478 (left) and Zn-sensitive inbred line Wu312 (right) under Zn-sufficient conditions at the 21th day after transfer. A 30 cm-length ruler was shown here.


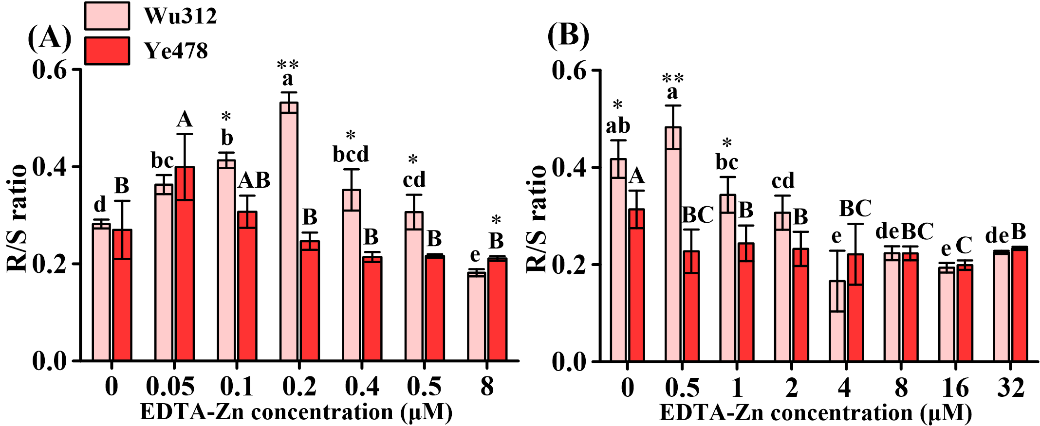


**Fig. S2** R/S ratios for Zn-sensitive inbred line Wu312 and Zn-tolerant inbred line Ye478 in Experiment 1 (A) and Experiment 2 (B). Different lowercase and upper letters indicate significant difference (*p* < 0.05) of Wu312 and Ye478 among treatments, respectively. * and ** indicate significant difference between Wu312 and Ye478 at *p* < 0.05 and *p* < 0.01, respectively.


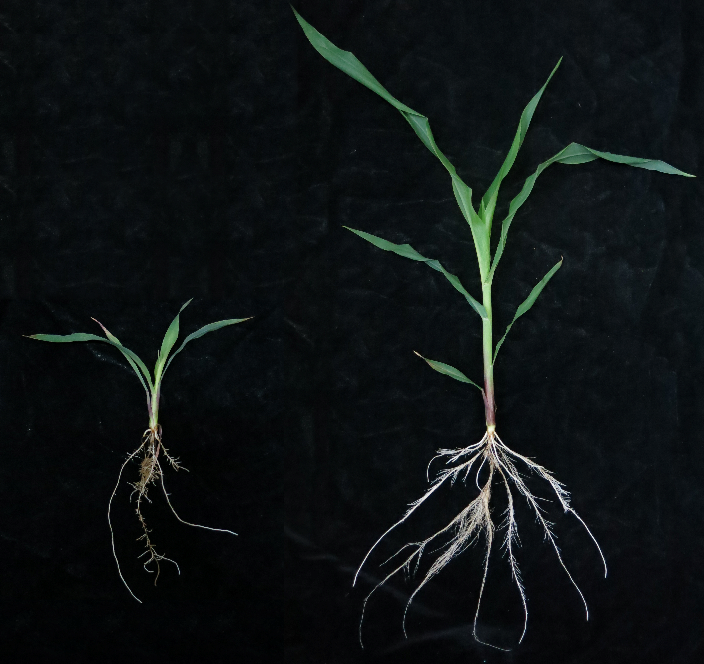


**Fig. S3** Shoots and roots of Zn-inefficient inbred line Wu312 (left) and Zn-efficient inbred line Ye478 (right) under Zn-deficient condition (0.5 μM).
